# Frank Abbott, M. D.

**Published:** 1897-06

**Authors:** 


### Frank Abbott, M. D.

Frank Abbott, M. D., died at his residence, 22 West 40th Street, New York City, April 20th, 1897. Dr. Abbott had been afflicted with heart trouble for the past two years, but only half an hour before his death his condition became serious.

Dr. Abbott was born in Shapleigh, Me., September 5th, 1836. He belonged to one of the oldest and best known families of New England. His ancestors came to America in 1640 and settled in Andover, Mass. Dr. Abbott attended school in his native town, and at about his twentieth year became a student in the office of a dentist of Oneida, N. Y. He afterwards removed to Johntown, N. Y., where he practiced his profession till he entered the army during the war, where he served as first lieutenant in Company E, of the 115 Regiment N. Y. Volunteers. After participating in several engagements he was taken prisoner at Harper's Ferry.

After the war Dr. Abbott removed to New York City, where he became an eminently successful dentist, and one of the bright lights of our profession. He graduated at the medical department of the University of the city of New York. In 1869 he was made dean, and professor of operative dentistry in the New York College of Dentistry, which position he held when he died. In 1876 he entered the laboratory of the late Dr. Carl Heitzman, where he worked diligently for many years. He has published many valuable papers, most of which he has collected in his last work " Dental Pathology and Practice."

Dr. Abbott was an enthusiast in all he undertook, he was fearless in speech, kind-hearted, and was highly esteemed by the majority of the dental profession. Dr. Abbott leaves a wife, two daughters and one son, Dr. Frank Abbott, Jr.

